# A case of isolated malrotation without midgut volvulus diagnosed prenatally and treated by laparoscopic surgery

**DOI:** 10.1186/s40792-024-02029-y

**Published:** 2024-09-26

**Authors:** Kosuke Endo, Hiroaki Fukuzawa, Yumi Mizoue, Atsushi Higashio, Mari Sonoda, Tamaki Iwade, Masahito Sato

**Affiliations:** Department of Pediatric Surgery, Kitano Hospital, The Tazuke Kofukai Medical Research Institute, 2-4-20 Ohgimachi, Kita-Ku, Osaka, 530-8480 Japan

**Keywords:** Malrotation, Midgut volvulus, Pediatric, Prenatal diagnosis, Laparoscopy

## Abstract

**Background:**

Malrotation is a congenital condition that predisposes individuals to midgut volvulus, which can result in significant bowel resection. While most cases of malrotation are diagnosed by the age of 1 year, typically presenting with symptoms related to volvulus or bowel obstruction, some cases remain asymptomatic. In children with visceral malposition, gastroschisis, omphalocele, or diaphragmatic hernia, malrotation may be suspected before symptoms manifest. However, isolated malrotation without midgut volvulus diagnosed prenatally is rare. We herein present a case of isolated malrotation without midgut volvulus that was prenatally diagnosed and successfully treated with laparoscopic surgery.

**Case presentation:**

A 30-year-old woman (gravida 3, para 1) underwent routine obstetric ultrasound, which revealed increased blood flow in the lower uterine segment and abnormal placental attachment. To rule out placenta percreta, magnetic resonance imaging was performed at 34 weeks of gestation. Incidentally, abnormal fetal intestinal arrangement was noted, with the colon localized in the left hemi-abdomen and the small intestine distributed in the right hemi-abdomen, raising suspicion of malrotation. Postnatal contrast studies confirmed the diagnosis of malrotation without midgut volvulus. Given the risk of midgut volvulus, a laparoscopic Ladd’s procedure was performed on day 6 of life. The postoperative course was uneventful, and the patient was still symptom-free 1 year postoperatively.

**Conclusions:**

This case illustrates that malrotation can be prenatally diagnosed using fetal magnetic resonance imaging. Considering the risk of midgut volvulus, prophylactic Ladd’s procedure should be performed in neonatal period. In cases where malrotation is not complicated by midgut volvulus, a laparoscopic Ladd procedure can be safely performed in neonates.

## Background

During embryonic development, the intestinal tract temporarily exits the abdominal cavity and is fully reintegrated around the 10th week of gestation. During this process, the intestine sufficiently elongates to meet the body’s needs. As it reenters the abdominal cavity, the intestine typically rotates 270 degrees counterclockwise and becomes anchored to the retroperitoneum. Malrotation is a congenital anomaly in which this rotation fails to occur properly, placing the intestine at risk for midgut volvulus. Most cases of malrotation are diagnosed within the first year of life, typically presenting with symptoms of volvulus or bowel obstruction, although some cases may remain asymptomatic. In children with associated conditions such as visceral malposition, gastroschisis, omphalocele, or diaphragmatic hernia, malrotation may be suspected prior to the onset of symptoms. However, isolated cases of malrotation without associated midgut volvulus diagnosed prenatally are exceedingly rare. We experienced a case of isolated malrotation without midgut volvulus that was prenatally diagnosed and treated laparoscopically.

## Case presentation

**Fig. 1:**
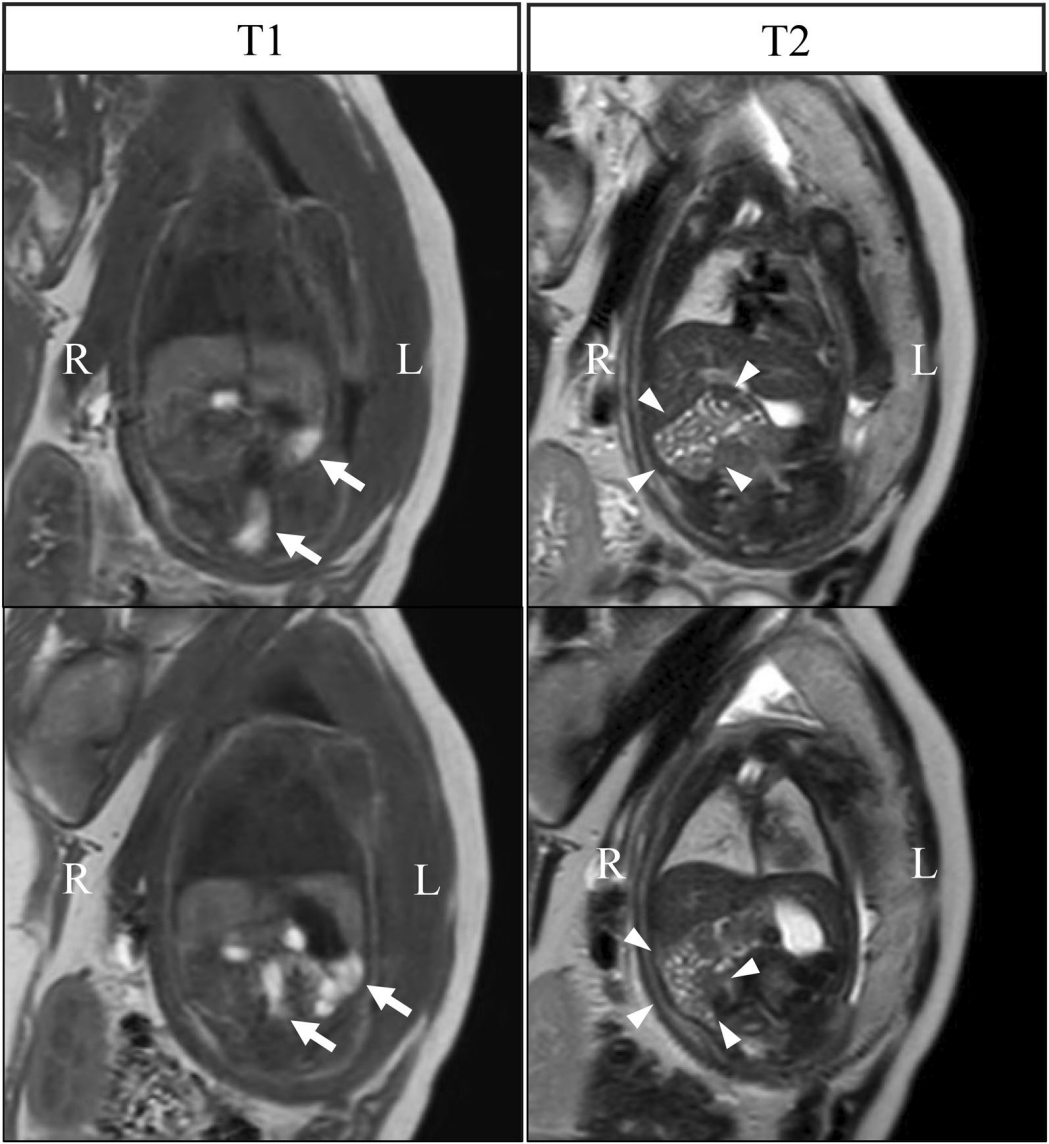
Fetal magnetic resonance imaging. The colon, visualized as hyperintense in the T1-weighted image, was distributed in the left hemi-abdomen (arrow). The small intestine, visualized as hyperintense in the T2-weighted image, was distributed in the right hemi-abdomen (arrowhead)

**Fig. 2:**
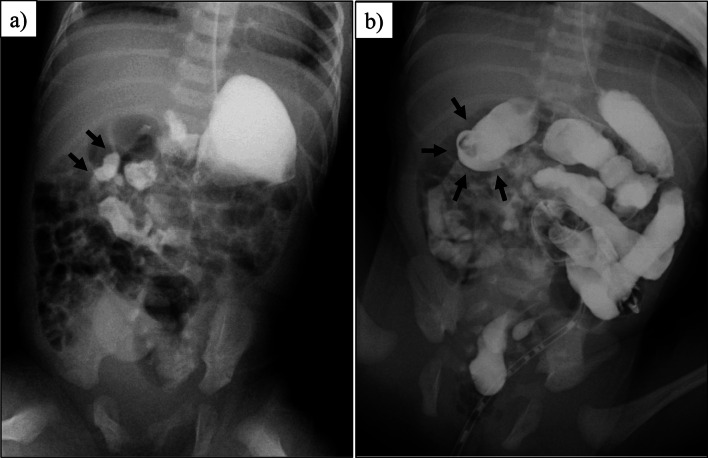
Postnatal contrast study. **a** Upper gastrointestinal study revealed that the Treitz ligament was not properly formed, and the first jejunal loop was located in the right-upper quadrant of the abdomen (arrow). **b** A contrast enema showed that the cecum was positioned in the right-upper abdomen (arrow)

A 30-year-old woman (gravida 3, para 1) underwent routine obstetric ultrasonography, showing increased blood flow in the lower uterine segment and abnormal attachment of the placenta (i.e., placenta previa). To exclude placenta percreta, magnetic resonance imaging (MRI) was performed at the 34th week of gestation. No apparent abnormalities were isolated in the placenta, but incidentally, abnormal arrangement of the fetal small intestine and colon was noted. T1-weighted imaging revealed that the colon, visualized as hyperintense, was localized in the left hemi-abdomen, and T2-weighted imaging showed the small intestine, also visualized as hyperintense, was distributed in the right hemi-abdomen. Malrotation was suspected (Fig. 1). The pregnancy progressed without incident, and a female neonate was born by scheduled cesarean section at 38 weeks 1 day with a birth weight of 2472 g and Apgar scores of 8/9. She was admitted to the neonatal intensive care unit for clinical observation. She passed meconium within the first 24 h, and no associated abnormalities were detected during screening tests. A gastrointestinal contrast study was performed on day 1 of life. Upper gastrointestinal imaging revealed that the Treitz ligament was not properly formed and that the first jejunal loop was located in the right-upper quadrant of the abdomen (Fig. 2a). Contrast media smoothly passed through the small intestine. A contrast enema showed that the cecum was in the right-upper abdomen (Fig. 2b). The diagnosis of malrotation was confirmed, and judging from the position of the cecum, the mesenteric base was suspected to be narrow, indicating a high risk of midgut volvulus. Surgery was performed on day 6 of life.

**Fig. 3:**
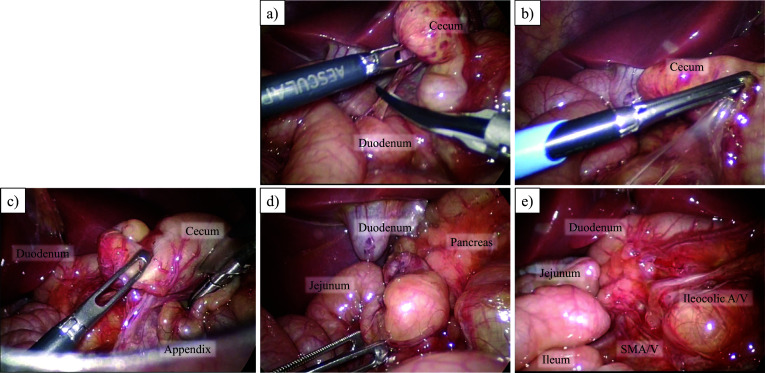
Laparoscopic findings. **a** Ladd’s band was dissected. **b**, **c** Accordingly, the abnormal membrane between the small intestine and cecum was dissected and widened the mesenteric root. Appendix was isolated in the right-upper quadrant of the abdomen. **d** Treitz ligament was not formed and the first jejunal loop was in the right-upper abdomen. **e** The mesentery was widened as much as possible. Ileocolic A/V: ileocolic artery and vein, SMA/V: superior mesenteric artery and vein

The operation was conducted laparoscopically. The patient was placed in the supine position, and three trocars were inserted at the umbilicus (5 mm), left upper abdomen (5 mm), and right flank (3.5 mm) with a pneumoperitoneum pressure of 8 mmHg. Laparoscopic observation confirmed that volvulus was not present. The intestine was not dilated, and sufficient space for the operation could be secured laparoscopically (Fig. 3a). Ladd’s ligament was dissected, and the mesentery was widened (Fig. 3b). The appendix was not resected. The operation time was 86 min, with minimal blood loss. The postoperative course was uneventful. Oral feeding commenced on postoperative day 1 and reached full volume by postoperative day 2. The patient was discharged on postoperative day 7. She remained in good condition and free from any symptoms for 1 year postoperatively.

## Discussion

Malrotation is typically diagnosed during the evaluation of bilious vomiting or abdominal pain, which are symptoms associated with complications such as midgut volvulus or bowel obstruction during the first year of life [1]. The estimated incidence of this condition is 1 in 500 births [2], but the true incidence is difficult to determine because some patients may remain asymptomatic throughout their lives. To date, only a few cases of prenatally diagnosed asymptomatic malrotation have been reported (Table 1) [3–6]. Cassart et al. [3] reported two cases of malrotation suspected on prenatal ultrasound based on medially positioned stomachs, which were confirmed postnatally. Biyyam et al. [4] described a case in which malrotation was suspected based on the finding of a midline stomach on fetal ultrasound and was confirmed by fetal MRI. Lesieur et al. [5] reported a fetus with dilated bowel loops in the left abdomen on prenatal ultrasound, and subsequent MRI confirmed the diagnosis of malrotation without volvulus. Zulli et al. [6] detailed a case in which fetal ultrasound showed a misplaced colon in the left abdomen and inversion of mesenteric vessels, with MRI confirming the diagnosis of malrotation. In our case, fetal MRI performed for another reason incidentally revealed the abnormal position of the intestine, leading to the diagnosis of malrotation without volvulus by contrast study after birth.

**Table 1:** Summary of the reports of prenatally diagnosed case of malrotation without midgut volvulus

Case	Author	Fetal US	Fetal MRI	Confirmation of the diagnosis	Treatment and outcome
1	Cassart^3)^	Medially positioned stomach	Not mentioned	Postnatal GI series	Observation, asymptomatic
2		Stomach positioned in the midline	Not mentioned	Postnatal upper GI contrast	Observation, asymptomatic
3	Biyyam^4)^	Midline stomach	Midline stomach, all loops of small bowel to the right of the midline and all large bowel to the left	Autopsy	Respiratory failure developed, died on the 12th day
4	Lesiur^5)^	Dilated bowel loops on the left abdomen	Dilatation was related to malpositioned colon that was entirely located in the left flank	Upper gastrointestinal series and barium enema	Observation, asymptomatic
5	Zulli^6)^	Large intestine misplaced in the left abdomen inversion of mesenteric vessels	The colon in the left abdomen and the small bowel in the right and central abdomen	Fetal MRI and postnatal contrast study	Ladd's operation (open), no event of volvulus
6	Our case	No abnormality was pointed out	The colon in the left hemi-abdomen, the small intestine in the right hemi-abdomen	Postnatal contrast studies	Laparoscopic Ladd's procedure, no event of volvulus

While the results of ultrasonography can be influenced by the operator’s skill and experience, MRI provides a more objective evaluation. Blask et al. [7] retrospectively evaluated cases of malrotation without obstruction that were confirmed by either imaging or surgery. They concluded that non-rotation of the bowel can indeed be detected using fetal MRI. Given the incidence of malrotation, which is approximately 1 in 500 births [2], it is likely that the prenatal diagnosis of isolated malrotation without midgut volvulus may become more common in the future.

Malrotation itself is not a life-threatening condition; however, when complicated by midgut volvulus, the situation becomes critical. Midgut volvulus can block blood flow to the intestine at the base of the superior mesenteric artery and vein, leading to ischemia of a significant portion of the intestine. If diagnosis is delayed, the resulting damage to the intestine can become irreversible, necessitating massive intestinal resection. This can lead to life-threatening conditions or result in short bowel syndrome. Therefore, prompt surgical intervention for symptomatic malrotation has gained widespread consensus as the appropriate treatment approach.

However, the treatment of asymptomatic malrotation remains controversial [8]. Given the potential risks associated with this condition, early diagnosis and treatment before the onset of symptoms related to midgut volvulus are ideal. Kedoin et al. [1] conducted a retrospective chart review of malrotation cases and found that 71.3% (57/80) of the cases involved neonates. Among these 57 neonates, midgut volvulus was observed in 41 and bowel resection was required in 4. Notably, in two of these four neonates, the torsion was only 180 degrees, highlighting that even mild volvulus can lead to critical intestinal ischemia in neonates. Additionally, 20 neonates (40.4%) did not present with bilious vomiting, illustrating the challenges in diagnosing symptomatic malrotation [1]. Malek et al. [9] used the data from the Nationwide Inpatient Sample and evaluated the role of the Ladd’s procedure in patients with asymptomatic malrotation. They reported that the gain in quality adjusted life expectancy associated with a prophylactic Ladd's procedure was highest when asymptomatic malrotation was treated at 1 year old and steadily declined until asymptomatic malrotation was treated at 20 years old. Covey et al. [10] compared pediatric patients who underwent a prophylactic Ladd procedure before any malrotation-related symptoms occurred (*n* = 19) with those who underwent the procedure after the onset of symptoms (*n* = 23). They found that the reoperation rate was significantly higher in the latter group, leading to the conclusion that prophylactic Ladd procedures are both safe and recommended [10]. However, when considering surgery, it is important to weigh the risks of postoperative complications, such as intestinal obstruction due to adhesions. Long-term follow-up studies have shown that postoperative complications are common in children with intestinal malrotation who undergo surgery [11, 12]. Mitsunaga et al. [12] reported that intestinal obstruction occurred in 22 of 87 cases (25.3%) following conventional open Ladd procedures.

A systematic review by the APSA Outcomes and Evidence-Based Practice Committee indicated that surgery may be considered for younger asymptomatic patients, whereas observation might be more appropriate for older asymptomatic patients [8]. Some reports suggest that the treatment strategy should be tailored according to the type of malrotation [5, 6]. Zulli et al. [6] argued that complete non-rotation can be managed conservatively because the mesenteric stalk is wide and the risk of volvulus is low. Conversely, incomplete rotation should be corrected to the position of non-rotation due to the narrow mesenteric root, which places the intestine at a significant risk of volvulus [6]. Lesieur et al. [5] emphasized that if a rotational disorder is suspected, it is crucial to eliminate incomplete rotation and assess the length of the mesenteric root by evaluating the positions of the angle of Treitz and the cecum. In our case, the contrast enema revealed that the cecum was located in the right-upper abdomen, and the mesenteric base was suspected to be narrow, indicating a high risk of midgut volvulus. We thoroughly explained to the parents the advantages and disadvantages of both surgery and observation. After considering the information provided, the parents opted for surgical intervention. In this case, the parents had been informed of the possibility of malrotation by the obstetrician in charge before the birth, so when we discussed the treatment about the patient, they could listen to our explanation calmly and make their decision smoothly.

The operation for malrotation was first described by Ladd [13] in 1936, and it has since become the gold standard surgical technique for this condition. The procedure involves detorsion of the volvulus, if present; division of the abnormal band between the cecum and duodenum (Ladd’s band); widening of the mesenteric root; and repositioning the intestine in a “non-rotation” position. The laparoscopic approach for treating malrotation was first reported by van der Zee [14] in 1995. Although the laparoscopic approach has gained favor because of better cosmetic outcomes, faster progression to full feeding, and shorter hospital stays, controversy remains regarding its efficacy, completeness, and the optimal patient population for this minimally invasive technique [15–19].

Compared with the open approach, the laparoscopic Ladd procedure generally has a lower rate of bowel obstruction but a higher rate of reoperation for re-volvulus. Zhu et al. [20] reported that the rate of postoperative re-volvulus is significantly higher following laparoscopic surgery than open surgery. However, Arnaud et al. [21] found that the rate of redo surgery was similar between the laparoscopic and open approaches. Reddy et al. [22] highlighted that the most frequent finding at reoperation was inadequate straightening of the duodenum, often attributed to poor visualization and local bleeding during the initial laparoscopic procedure.

Another issue with the laparoscopic approach is the high conversion rate to open surgery. Most children with this condition present with symptoms and undergo surgery within the first year of life, making the laparoscopic procedure technically challenging and requiring a high level of surgical skill. Consequently, the conversion rate to open surgery is relatively high. Miyano et al. [23] reported that the laparoscopic Ladd procedure cannot be recommended for the treatment of malrotation in neonates. By contrast, Xie et al. [24] compared the laparoscopic Ladd procedure with the open approach and concluded that the former is a safe option, even in infants and neonates, provided it is performed at a center with expertise in laparoscopic surgery. Arnaud et al. [21] also noted that conversions from laparoscopic to open surgery occurred more frequently during the early stages of a surgeon’s experience and emphasized that the procedure should be performed by an expert. In our case, malrotation was suspected prenatally, and the diagnosis was confirmed early, before the occurrence of volvulus. The operation was performed as scheduled, allowing for excellent laparoscopic visualization. The intestines were neither dilated nor edematous, which facilitated the safe dissection of Ladd’s ligament and sufficient widening of the mesentery. We believe that the laparoscopic Ladd procedure can be performed safely, even in neonates, under elective circumstances where midgut volvulus is not complicated.

## Conclusions

We encountered a rare case of prenatally diagnosed isolated malrotation without midgut volvulus. Considering the risk of midgut volvulus, prophylactic Ladd’s procedure should be performed electively in neonates if diagnosed prenatally. In situations where midgut volvulus is not present, the laparoscopic Ladd procedure can be safely performed, even in neonates.

## Data Availability

The datasets used and/or analyzed during the current study are available from the corresponding author on reasonable request.

## Abbreviation

MRI: Magnetic resonance imaging

## References

[1] Kedoin C, Muto M, Nagano A, Matsui M, Sugita K, Baba T, et al. Notable clinical differences between neonatal and post-neonatal intestinal malrotation: a multicenter review in southern Japan. J Pediatr Surg. 2024;59:566–70. 38145920 10.1016/j.jpedsurg.2023.11.020

[2] Torres AM, Ziegler MM. Malrotation of the intestine. World J Surg. 1993;17:326–31. 8337878 10.1007/BF01658699

[3] Cassart M, Massez A, Lingier P, Absil AS, Donner C, Avni F. Sonographic prenatal diagnosis of malpositioned stomach as a feature of uncomplicated intestinal malrotation. Pediatr Radiol. 2006;36:358–60. 16465538 10.1007/s00247-005-0074-1

[4] Biyyam DR, Dighe M, Siebert JR. Antenatal diagnosis of intestinal malrotation on fetal MRI. Pediatr Radiol. 2009;39:847–9. 19333589 10.1007/s00247-009-1226-5

[5] Lesieur E, Lecompte JF, Gorincour G, Potier A, Héry G, Bretelle F, et al. Prenatal diagnosis of complete nonrotation of fetal bowel with ultrasound and magnetic resonance imaging. Diagn Interv Imaging. 2016;97:687–9. 26837854 10.1016/j.diii.2016.01.003

[6] Zulli A, Tocchioni F, Oreglio C, Biagiotti R, Di-Maurizio M, Morini F. Prenatal diagnosis of isolated bowel malrotation and its impact on post-natal management A case report and review of the literature. J Pediatr Surg Case Reports. 2023;92: 102627

[7] Blask AR, Fagen KE, Rubio EI, Badillo AT, Bulas DI. Prenatal diagnosis of intestinal nonrotation using magnetic resonance imaging: Is it possible? Pediatr Radiol. 2021;51:1332–8. 33608743 10.1007/s00247-021-04969-1

[8] Graziano K, Islam S, Dasgupta R, Lopez ME, Austin M, Chen LE, et al. Asymptomatic malrotation: diagnosis and surgical management: An American Pediatric Surgical Association outcomes and evidence based practice committee systematic review. J Pediatr Surg. 2015;50:1783–90. 26205079 10.1016/j.jpedsurg.2015.06.019

[9] Malek MM, Burd RS. The optimal management of malrotation diagnosed after infancy: a decision analysis. Am J Surg. 2006;191(1):45–51. 16399105 10.1016/j.amjsurg.2005.10.002

[10] Covey SE, Putnam LR, Anderson KT, Tsao K. Prophylactic versus symptomatic Ladd procedures for pediatric malrotation. J Surg Res. 2016;205:327–30. 27664880 10.1016/j.jss.2016.06.097

[11] Murphy FL, Sparnon AL. Long-term Complications following intestinal malrotation and the Ladd’s procedure: a 15 year review. Pediatr Surg Int. 2006;22:326–9. 16518597 10.1007/s00383-006-1653-4

[12] Mitsunaga T, Saito T, Terui K, Nakata M, Ohno S, Mise N, et al. Risk factors for intestinal obstruction after Ladd Procedure. Pediatr Rep. 2015;7:5795. 26266030 10.4081/pr.2015.5795 PMC4508621

[13] Ladd W. Surgical diseases of the alimentary tract in infants. N Engl J Med. 1936;17:705–8.

[14] Zee DC, Bax NM. Laparoscopic repair of acute volvulus in a neonate with malrotation. Surg Endosc 1995;9:1123–4 10.1007/BF00189001 8553217

[15] Saberi RA, Gilna GP, Slavin BV, Cioci AC, Urrechaga EM, Parreco JP, et al. Outcomes for Ladd’s procedure: does approach matter? J Pediatr Surg. 2022;57:141–6. 34657741 10.1016/j.jpedsurg.2021.09.016

[16] Ferrero L, Ahmed YB, Philippe P, Reinberg O, Lacreuse I, Schneider A, et al. Intestinal malrotation and volvulus in neonates: laparoscopy versus open laparotomy. J Laparoendosc Adv Surg Tech A. 2017;27:318–21. 28055334 10.1089/lap.2015.0544

[17] Catania VD, Lauriti G, Pierro A, Zani A. Open versus laparoscopic approach for intestinal malrotation in infants and children: a systematic review and meta-analysis. Pediatr Surg Int. 2016;32:1157–64. 27709290 10.1007/s00383-016-3974-2

[18] Huntington JT, Lopez JJ, Mahida JB, Ambeba EJ, Asti L, Deans KJ, et al. Comparing laparoscopic versus open Ladd’s procedure in pediatric patients. J Pediatr Surg. 2017;52:1128–31. 27856011 10.1016/j.jpedsurg.2016.10.046

[19] Zhang Z, Chen Y, Yan J. Laparoscopic versus open Ladd’s procedure for intestinal malrotation in infants and children: a systematic review and meta-analysis. J Laparoendosc Adv Surg Tech A. 2022;32:204–12. 34609912 10.1089/lap.2021.0436

[20] Zhu H, Zheng S, Alganabi M, Peng X, Dong K, Pierro A, et al. Reoperation after Ladd’s procedure in the neonatal period. Pediatr Surg Int. 2019;35:117–20. 30382377 10.1007/s00383-018-4382-6

[21] Arnaud AP, Suply E, Eaton S, Blackburn SC, Giuliani S, Curry JI, et al. Laparoscopic Ladd’s procedure for malrotation in infants and children is still a controversial approach. J Pediatr Surg. 2019;54:1843–7. 30442460 10.1016/j.jpedsurg.2018.09.023

[22] Reddy AS, Shah RS, Kulkarni DR. Laparoscopic Ladd’s Procedure in Children: challenges, results, and problems. J Indian Association Pediatr Surg. 2018;23:61–5. 10.4103/jiaps.JIAPS_126_17 PMC5898205 29681694

[23] Miyano G, Fukuzawa H, Morita K, Kaneshiro M, Miyake H, Nouso H, et al. Laparoscopic repair of malrotation: what are the indications in neonates and children? J Laparoendosc Adv Surg Tech A. 2015;25:155–8. 25647302 10.1089/lap.2014.0236

[24] Xie W, Li Z, Wang Q, Wang L, Pan Y, Lu C. Laparoscopic vs open Ladd’s procedure for malrotation in neonates and infants: a propensity score matching analysis. BMC Surg. 2022;22:25. 35081938 10.1186/s12893-022-01487-1 PMC8793198

